# Histological skeletochronology indicates developmental plasticity in the early Permian stem lissamphibian *Doleserpeton annectens*

**DOI:** 10.1002/ece3.6054

**Published:** 2020-02-06

**Authors:** Bryan M. Gee, Yara Haridy, Robert R. Reisz

**Affiliations:** ^1^ Department of Biology University of Toronto Mississauga Mississauga ON Canada; ^2^ Leibniz‐Institut für Evolutions‐ und Biodiversitätsforschung Museum für Naturkunde Berlin Germany; ^3^ International Centre for Future Science Dinosaur Evolution Research Center Jilin University Changchun China

**Keywords:** Amphibamiformes, development, histology, plasticity, Temnospondyli

## Abstract

*Doleserpeton annectens* is a small‐bodied early Permian amphibamiform, a clade of temnospondyl amphibians regarded by many workers to be on the lissamphibian stem. Most studies of this taxon have focused solely on its anatomy, but further exploration of other aspects of its paleobiology, such as developmental patterns, is critical for a better understanding of the early evolutionary history of lissamphibians. Here, we present a histological analysis of growth patterns in *D. annectens* that utilizes 60 femora, the largest sample size for any Paleozoic tetrapod. We identified pervasive pairs of closely spaced lines of arrested growth (LAGs), a pattern that indicates a marked degree of climatic harshness and that would result in two cessations of growth within a presumed single year. We documented a wide degree of variation compared to previous temnospondyl skeletochronological studies, reflected in the poor correlation between size and inferred age, but this observation aligns closely with patterns observed in extant lissamphibians. Furthermore, sensitivity analyses conducted by subsampling our dataset at more typical sample sizes for paleontological studies produced a wide range of results. This includes biologically improbable results and exceptionally well‐fit curves that demonstrate that low sample size can produce potentially misleading artifacts. We propose that the weak correlation between age and size represents developmental plasticity in *D. annectens* that typifies extant lissamphibians. Detection of these patterns is likely only possible with large sample sizes in extinct taxa, and low sample sizes can produce false, misleading results that warrant caution in drawing paleobiological interpretations from such samples.

## Graphical abstract

This manuscript presents a skeletochronological examination of growth patterns of the early Permian stem lissamphibian *Doleserpeton annectens*. Through the largest sample size for any Paleozoic tetrapod (*n* = 60), we identify patterns of developmental plasticity in response to climatic harshness and seasonality that mirror the patterns seen in crown lissamphibians.

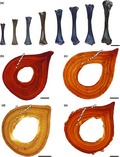

## 1 INTRODUCTION

*Doleserpeton annectens* Bolt, 1969 is an early Permian amphibamiform, a clade of small‐bodied dissorophoid temnospondyls that has been frequently hypothesized to be closely related to some (e.g., Anderson, Reisz, Scott, Fröbisch, & Sumida, 2008; Pardo, Small, & Huttenlocker, 2017) or all (e.g., Bolt, 1969; Schoch, 2019; Sigurdsen & Bolt, 2010) of the lissamphibian crown groups (but see Marjanović & Laurin, 2013, 2019 for an advocacy of a monophyletic origin within the lepospondyls). *Doleserpeton annectens* is known only from the karst deposits near Richards Spur, Oklahoma, where it occurs as part of the diverse tetrapod assemblage interpreted to represent an upland ecosystem (MacDougall, Tabor, Woodhead, Daoust, & Reisz, 2017). The long‐bone histology of *D. annectens* has been previously examined (e.g., Castanet, Francillon‐Vieillot, de Ricqlés, & Zylberberg, 2003), but this is the first skeletochronological study across a broad range of sizes (and inferred semaphoronts). More broadly, the long bones of temnospondyls have been previously studied to identify patterns of growth at the histological level (e.g., de Ricqlés, 1981; McHugh, 2014, 2015; Mukherjee, Ray, & Sengupta, 2010; Sanchez, de  Ricqlès, Schoch, & Steyer, 2010; Sanchez & Schoch, 2013; Sanchez, Steyer, Schoch, & de Ricqlès, 2010), but the few studies involving relatively large sample sizes with reconstructed growth series have focused primarily on large‐bodied, aquatic stereospondyls (Konietzko‐Meier & Klein, 2013; Konietzko‐Meier & Sander, 2013; Steyer, Laurin, Castanet, & de Ricqlès, 2004).

**Figure 1:**
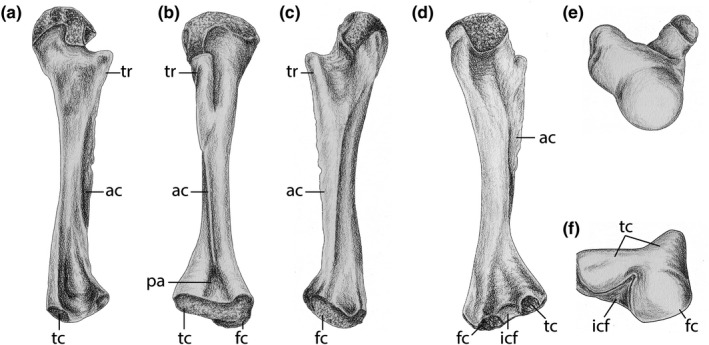
Illustration of a generalized femur of *Doleserpeton annectens*. (a) Anterior view, (b) flexor view, (c) posterior view, (d) extensor view, (e) proximal view, and (f) distal view. Landmark features are labeled; abbreviations: ac, adductor crest; fc, fibular condyle; icf, intercondylar fossa; pa, popliteal area; tc, tibial condyle; tr, trochanter. Artwork: P. Urban

To date, only two other amphibamiforms have been histologically sampled, the early Permian branchiosaurid *Apateon* von Meyer, 1844 spp. from Europe (Sanchez, de Ricqlès, et al., 2010; Sanchez, Steyer, et al., 2010) and the Early Triassic micropholid *Micropholis stowi* Huxley, 1859 from South Africa (McHugh, 2015), and neither study provided quantitative assessments of growth. The paucity of work on amphibamiforms stems from the paucity of material; many terrestrial amphibamiforms are represented by few specimens and predominantly cranial material that is not amenable to histological sampling. *Doleserpeton annectens* is thus unique in being one of the most abundant tetrapods at the Richards Spur locality, rivaled only by the eureptile *Captorhinus* Cope, 1895 (MacDougall, 2017), with dense accumulations of *Doleserpeton*‐bearing matrix referred to as “D‐concentrate” (Bolt, 1969). In particular, it is well‐represented by long bones, more specifically femora (Figure 1). Therefore, the abundance of material, in concert with the highly nested position of *D. annectens* within Amphibamiformes (e.g., Schoch, 2019; Sigurdsen & Bolt, 2010), makes this taxon an ideal candidate for exploring aspects of developmental paleobiology along the presumed lissamphibian stem. The objectives of this study are twofold: (a) to compile and to examine the skeletochronology of *D. annectens* using long bone histology and (b) to assess the effect of sample size on skeletochronological studies through the use of sensitivity analyses by subsampling the exceptionally large sample size (*n* = 60) available for this taxon.

## 2 METHODS

### 2.1 Specimen selection

**Figure 2:**
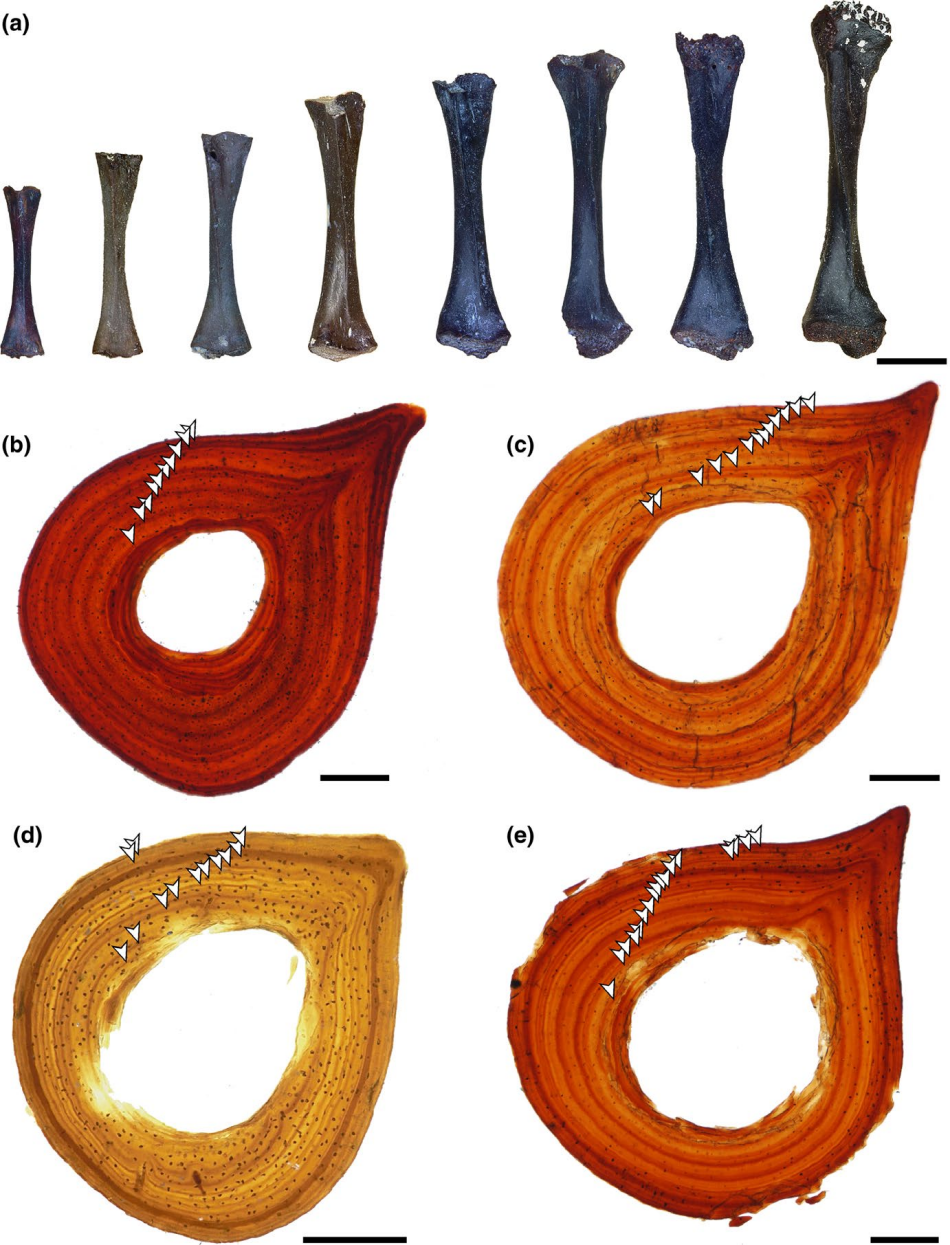
Photographs of whole‐element femora and histological thin sections. (a) Representative size range of femora sampled in this study. (b) ROMVP 80680 (double LAG pattern). (c) ROMVP 80676 (indeterminate pattern). (d) ROMVP 79319 (single LAG pattern). (e) ROMVP 79337 (double LAG pattern). Arrows represent lines of arrested growth (LAGs). Scale bars: 2 mm (a); 0.25 mm (b–e)

In order to examine growth patterns in *D. annectens*, sixty isolated femora from the early Permian karst deposits near Richards Spur, Oklahoma, that were donated by W. May (Figures 1 and 2). Embedded specimen resin blocks and their associated thin sections are reposited at the Royal Ontario Museum Vertebrate Paleontology collection (ROMVP) and are assigned the catalogue numbers of ROMVP 79310 to 79354, inclusive, and ROMVP 80676 to 80690, inclusive (Table 1).

**Table 1:** Datasheet with measurements, LAG counts, and adjusted LAG counts for specimens sampled in this study

Specimen number	Length (mm)	Observed LAGs	Inferred age (years)	Retrocalculated age (years)	Pattern
79310	7.97	15	8	9	Double
79311	8.42	17	9	10	Double
79312	9.36	15	12	13	Indeterminate
79313	8.88	17	10	11	Double ending in single
79314	8.12	16	8	9	Double
79315	6.36	10	5	5	Double
79316	6.09	8	4	4	Double
79317	6.59	4	4	4	Single
79318	5.9	4	4	4	Single
79319	6.33	12	12	13	Single
79320	8.15	10	5	6	Double
79321	7.61	10	5	6	Double
79322	8.16	10	5	5	Double
79323	7.17	11	7	8	Double ending in single
79324	7.4	11	11	11	Single
79325	7.42	14	7	8	Double
79326	6.98	4	4	4	Single
79327	7.06	10	7	8	Double ending in single
79328	7.45	8	8	8	Single
79329	7.23	10	5	6	Double
79330	7.3	12	8	9	Double ending in single
79331	6.4	6	3	4	Double
79332	6.75	8	4	5	Double
79333	6.81	8	4	5	Double
79334	7.25	8	4	5	Double
79335	7.93	11	7	8	Double ending in single
79336	8.9	10	5	6	Double
79337	8.59	15	9	10	Double ending in single
79338	8.26	11	6	7	Double
79339	9.35	17	9	10	Double
79340	10.12	17	11	12	Double ending in single
79341	9.72	15	8	9	Double
79342	9.82	16	8	9	Double
79343	9.86	16	8	9	Double
79344	8.79	14	7	8	Double
79345	6.22	5	4	4	Double ending in single
79346	6.13	6	3	3	Double
79347	6.27	6	3	3	Double
79348	6.69	10	5	6	Double
79349	5.79	—	—	—	?
79350	5.99	7	4	4	Double
79351	6.67	—	—	—	?
79352	6.14	—	—	—	?
79353	6.54	10	5	5	Double
79354	6.36	16	8	9	Double
80676	8.79	12	9	10	Indeterminate
80677	9.06	17	8	9	Indeterminate
80678	9.1	15	8	9	Indeterminate
80679	9.79	11	6	7	Double
80680	9.19	11	6	7	Double
80681	6.42	10	5	5	Double
80682	6.71	—	—	—	?
80683	8.14	—	—	—	?
80684	7.41	—	—	—	?
80685	6.88	9	5	5	Double
80686	6.22	10	5	6	Double
80687	4.88	8	4	4	Double
80688	5.91	—	—	—	?
80689	5.84	—	—	—	?
80690	5.92	11	6	7	Double

Note

An indeterminate pattern is one that was not clearly consistent (e.g., only double LAGs) or unidirectional (e.g., double LAGs ending with single LAGs).

Specimen identification was based on previous descriptions of the femur of *D. annectens* (e.g., Sigurdsen & Bolt, 2010). Femora were selected based on completeness and to sample as broad of a size range as possible. Only right femora were chosen to avoid potential sampling of the same individual. General features of terrestrial dissorophoid femora are conserved, but relative osteological development and size‐related features make taxonomic distinctions from the large‐bodied olsoniforms readily apparent (e.g., Sullivan, Reisz, & May, 2000). However, we note that there is virtually no postcrania (and no limb elements) of the other two Richards Spur amphibamiforms, *Pasawioops mayi* Fröbisch & Reisz, 2008 and *Tersomius dolesensis* Anderson & Bolt, 2013. Based on the current understanding of size relationships, *P. mayi* appears to have reached a much larger adult size than *Doleserpeton*. The holotype skull measures 32.6 mm in length (Fröbisch & Reisz, 2008), but much larger individuals have also been reported (Maddin, Fröbisch, Evans, & Milner, 2013), while *D. annectens* skulls range from 12 to 19 mm (Bolt, 1969; Sigurdsen & Bolt, 2010). *T. dolesensis* is somewhat larger than *D. annectens*; the holotype skull of the former is about 22.5 mm in length, but its level of ossification indicates that it may not be a full adult (Anderson & Bolt, 2013). Material referred to *D. annectens* is far more abundant than *P. mayi* and *T. dolesensis* (Bolt, 1969; Sigurdsen & Bolt, 2010) and further comments on how this affects the results of this study are presented in the discussion.

### 2.2 Histological preparation

Histological sampling followed standard procedures of the Royal Ontario Museum (ROM). Specimens were photographed in several standard profiles using a Leica DVM6 tilting microscope prior to sampling. Specimens were glued to a base coat to standardize sectioning plane, embedded using Castolite AC resin, allowed to cure for a minimum of 24 hr, and sectioned at approximately the minimum diaphyseal circumference, which does not necessarily correspond to the geometric mid‐length of the element. Because of the small size of these specimens, the cut was made slightly to one side of this minimum circumferential region in order to avoid a kerf loss (0.25 mm) that could result in the full loss of the minimum circumference. Specimens were cut using a Buehler IsoMet 1000 fitted with a 0.25‐mm wafer blade at a speed of 275 rpm and then mounted to frosted plexiglass slides using cyanoacrylate adhesive. Polishing was done using the Hillquist 1010 grinding cup, followed by manual polishing on glass plates with 1,000‐mesh levigated grit and 5‐micron aluminum dioxide. Slides were imaged on a Leica DVM6 digital transmitted light microscope using LAS (Leica Application Suite) X software and a Nikon AZ‐100 microscope with a Nikon DS‐Fi2 camera using NIS Elements BR software. LAG counting was performed under microscope rather than from captured microphotographs.

### 2.3 Skeletochronological methods

**Figure 3:**
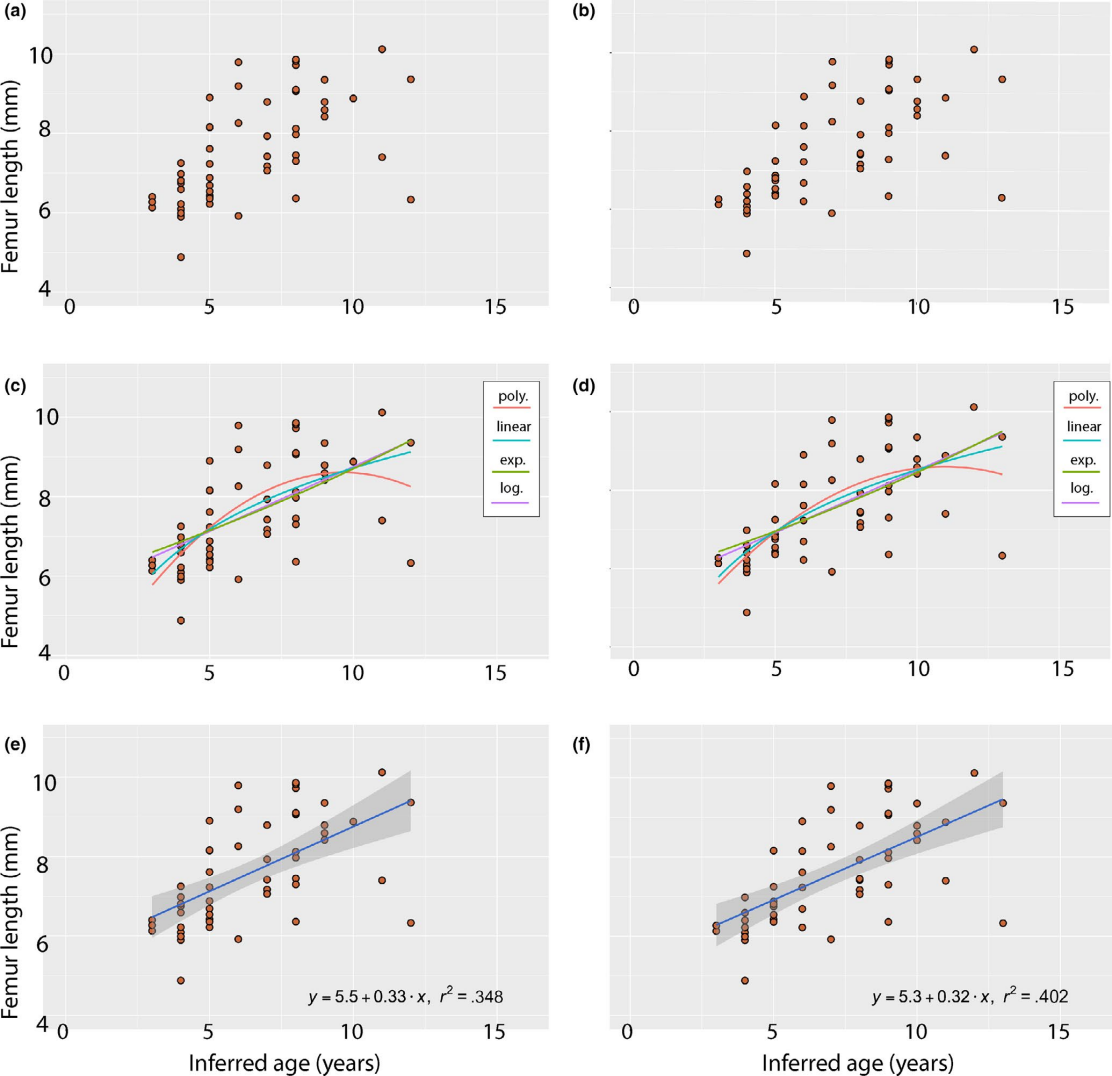
Distribution of data points of all specimens for which LAGs could be counted (*n* = 52) plotted against femur length. (a) Data plot of age inferred from observational counts (raw data) versus femur length. (b) The same for the age inferred from retrocalculated counts (adjusted data). (c) Comparison of models fit to the raw data. (d) The same for the adjusted data. (e) Simple linear model fit for the raw data with 95% confidence intervals in dark grey. (f) The same for the adjusted data. Legend abbreviations: poly. = second‐order polynomial; linear = simple linear; exp. = exponential; log. = logarithmic

Retrocalculation methods (Woodward, Padian, & Lee, 2013) are often used to infer the amount of skeletochronological information that has been lost due to remodeling of the periosteal bone from within the medullary cavity. Some of the most common methods include section stacking, in which sections of variable size (and presumably variable maturity) are overlaid to infer how many growth cycles have been obscured, and arithmetic models in which the spacing between lines of arrested growth (LAGs) in variably sized specimens is measured and then combined to reconstruct a section with a fully preserved record. Both methods rely on a consistent plane of section at the minimum diaphyseal diameter. This is typically not a major challenge for large‐bodied tetrapods in which an entire “puck” containing the minimum diaphyseal region can be extracted and an exact section can be made. However, sectioning small elements that are sometimes less than 5 mm in the longest axis while maintaining a perfectly consistent plane is challenging. As a result, there are clear differences between sections related to the relative cortical thickness and medullary cavity circumference. This does not affect the quality or counting of LAGs when sections are made approximately at this region (personal observation). However, it does interfere with the aforementioned methods in which a perfectly consistent plane is required to properly align growth marks and to properly compare relative cortical thickness. In order to at least account for specimens with the same LAG count, but with disparity in the presence of endosteal remodeling (suggesting that ones with remodeling are older than their LAG count indicates), we added one LAG to each specimen in which remodeling was observed. Both adjusted and nonadjusted data are presented in the results (Figure 3). This is admittedly semi‐arbitrary, as some individuals almost certainly lost more than one annual growth cycle to remodeling, but we elected to pursue a more conservative approach given the lack of reliability of retrocalculation methods for this dataset. Without any sort of calibration method similar to mark–recapture studies performed in extant tetrapods, it is unclear at which point more than one LAG may be lost to remodeling, and we did not attempt to define an arbitrary size or inferred age threshold at which to add a second inferred growth cycle.

Double LAGs were counted as one LAG in the skeletochronological analysis. This is based on previous work on both extinct (e.g., Sanchez, Steyer, et al., 2010) and extant (e.g., Smirina, 1994) studies in which double LAGs are interpreted as a biannual cessation of growth, often (but not necessarily) during the most unfavorable seasons of the year. Double LAGs were identified by the presence of two very closely spaced LAGs whose spacing was distinctly narrower than the adjacent LAGs on either side. Thus, the absolute spacing between pairs of LAGs could be nearly identical, but the determination of single versus double LAGs was made by comparison of the spacing relative to adjacent LAGs. A table with specimen numbers, measurements and LAG counts is included above (Table 1). Various model fits were attempted for the retrocalculated data set. All data analysis was performed in R Studio v.1.2.1335.

### 2.4 Sensitivity analysis

In order to assess the effect of sample size on growth curve reconstructions, we performed a random subselection experiment. Specimens were randomly sampled (repetition, or selection of the same specimen, was prohibited) in bins of 10, 20, and 30 samples, and then, a simple linear regression (*y* = *mx* + *b*) was computed. Under this model, the y‐intercept (b) represents the hypothetical femoral length at birth (time = 0), although the femur is virtually assured not to have been fully ossified at this time, and the slope (m) represents the “growth rate” in the sense of change in femur length relative to the proxy for age (LAG count). This model was selected because it has been used in other studies and because the goal of this analysis is only to test the effects of sample size variation, rather than to conclusively determine the best model or to refine an existing one. This was run for 5,000 iterations for each sampling bin. Histograms for the correlation coefficient (*R* ^2^ value), the y‐intercept, and the slope were then produced. This analysis was run using the retrocalculated data. All data analysis was performed in R Studio v.1.2.1335.

## 3 RESULTS

### 3.1 Histological description and ontogeny

The bone histology and microanatomy of *Doleserpeton* Bolt, 1969 has been analyzed and described by previous workers (Castanet et al., 2003; de Ricqlés, 1981; Laurin, Girondot, & Loth, 2004) and are only briefly readdressed here. The histological structures are relatively simple, with lamellar bone sequentially deposited at the periosteal surface, and resorbed and replaced in older individuals by endosteal lamellar bone at the boundary of the medullary cavity (Figure 2). Discrete lines of arrested growth (LAGs) extend around the entire circumference of the femur, although in some remodeled areas, only part of a line remains. In almost all specimens, double LAGs, which manifest as closely spaced lines separated from the next LAG(s) by a broader band of bone deposition, were identified and counted as a single LAG. A few specimens with other patterns were also noted; these comprise single LAGs (*n* = 6), double LAGs transitioning to single LAGs toward the periphery (i.e., later in development; *n* = 7), and an indeterminate pattern without a discernible trend (*n* = 4). The sample size for these uncommon LAG patterns is too low to derive reasonable quantitative correlations with size regardless of the distribution of the four points; data plotted by LAG pattern are included in Figure S1. Full‐size microphotographs of representative thin sections are included in Appendix 1.

Similar variation was noted in the study of the branchiosaurid *Apateon* by Sanchez, Steyer, et al. (2010), although those patterns may be more readily correlated with the variable paleoaltitudes of montane lakes (but see Laurin & Soler‐Gijón, 2010; Schultze, 2009 for alternative paleoenvironmental interpretations). In contrast, LAGs are totally absent in a sampled humerus of the terrestrial amphibamiform *M. stowi*. This was interpreted as evidence for fast growth during early development among Early Triassic temnospondyls (McHugh, 2015), although in the strictest sense, it indicates only that growth did not totally cease in this taxon. The presence of fibrolamellar bone in *M. stowi* (rather than lamellar bone as in *Apateon* and *D. annectens*) suggests a faster absolute growth rate that is likely associated with its larger size (Schoch & Rubidge, 2005).

Remodeling is variable across our sample, and secondary osteons are not present, as in *Apateon* and *M. stowi* (McHugh, 2015; Sanchez, Steyer, et al., 2010). Some specimens of *D. annectens* have a relatively thick layer of endosteal bone that is separated from the periosteal lamellar bone by a line of resorption and that extends around the entire circumference of the medullary cavities. Other specimens only have incomplete rings of remodeling. Osteocyte lacunae are relatively evenly dispersed throughout the cortex, although they cannot always be clearly discerned because they appear to be translucent or are not visible in the focal plane of the thin section when sections are not z‐stacked.

### 3.2 Growth curve reconstruction

LAGs could be definitively counted for 52 of the 60 sections that were produced (Table 1). The data were plotted and then attempts were made to fit various linear models (simple linear, second‐order polynomial, exponential, logarithmic) to the data (Table 1; Figure 3c,d). Although growth curves are rarely accurately modeled by simple linear models, particular stages of development may be approximated by these models. All of these models produced poor fits (compared to other temnospondyl studies) on both the adjusted and the nonadjusted datasets, with R^2^ (correlation coefficient) values all below 0.500 (Table 2); this is distinctly lower than previous studies (e.g., Konietzko‐Meier & Sander, 2013; Steyer et al., 2004) in which the *R* ^2^ values exceeded 0.9. For all models, the coefficient values increased slightly with the adjusted (retrocalculated) dataset. The best fit model was the second‐order polynomial model, but arguably this is the most biologically improbable growth curve, as it forms a convex parabola. The simple linear and exponential models were essentially identical. *p*‐Values for all models (calculated via a Wald test), for both adjusted and nonadjusted data, were well below the typical .05 threshold for determining statistical significance and would round to .000 with three significant figures. Due to the spread of the data and the observation that our sample captures an incomplete representation of the entire growth trajectory, attempting to fit the data using nonlinear growth models (Gompertz, von Bertalanffy [VBGM], sigmoidal) was not possible. There are no patterns in the data that indicate dimorphism, which could be variably ascribed to a number of difficult‐to‐test attributes such as sexual dimorphism, distinct populations (or taxa), or directional evolution.

**Table 2:** Comparison of linear model fits for nonadjusted (observed) and adjusted (retrocalculated) datasets

Model	Equation (obs.)	R^2^ (obs.)	Equation (adj.)	R^2^ (adj.)
Linear	*y* = 0.326 × *x* + 5.497	.348	*y* = 0.318 × *x* + 5.326	.402
Second‐order polynomial	*y* = −0.064 × *x* ^2^ + 1.24 × *x* + 2.6305	.473	*y* = −0.047 × *x* ^2^ + 1.034 × *x* + 2.934	.470
Logarithmic	*y* = 2.221 × ln(*x*) + 3.606	.395	*y* = 2.733 × ln(*x*) + 3.286	.443
Exponential	*y* = 5.678e^0.043 × ^ *^x^*	.347	*y* = 5.546e^0.042 × ^ *^x^*	.404

### 3.3 Sensitivity analysis

**Figure 4:**
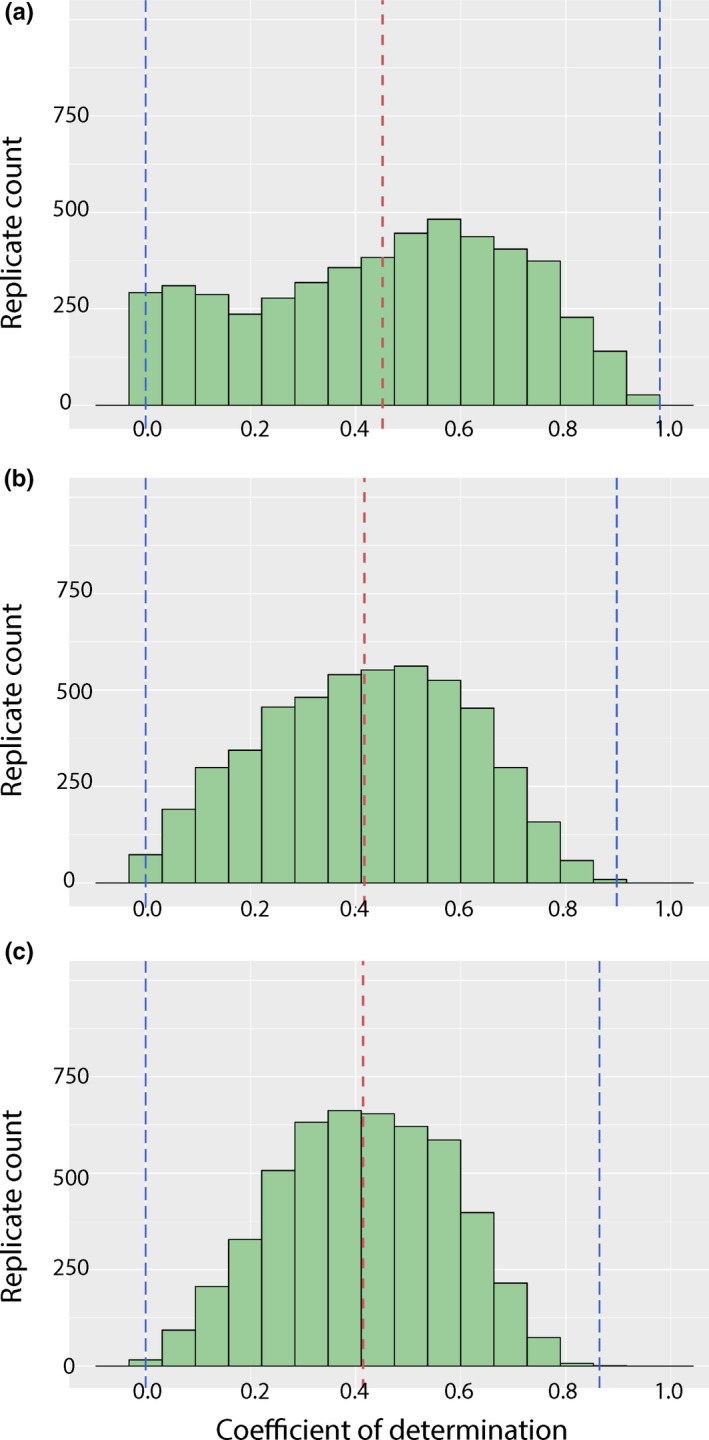
Histograms showing effect of sample size on variability in estimates of correlation (*r* ^2^) between size and individual age. (a) Distribution of values with sampling bin of 10. (b) Distribution of values with sampling bin of 20. (c) Distribution of values with sampling bin of 30. Red lines demarcate the mean; blue lines demarcate the minimum and maximum values

**Figure 5:**
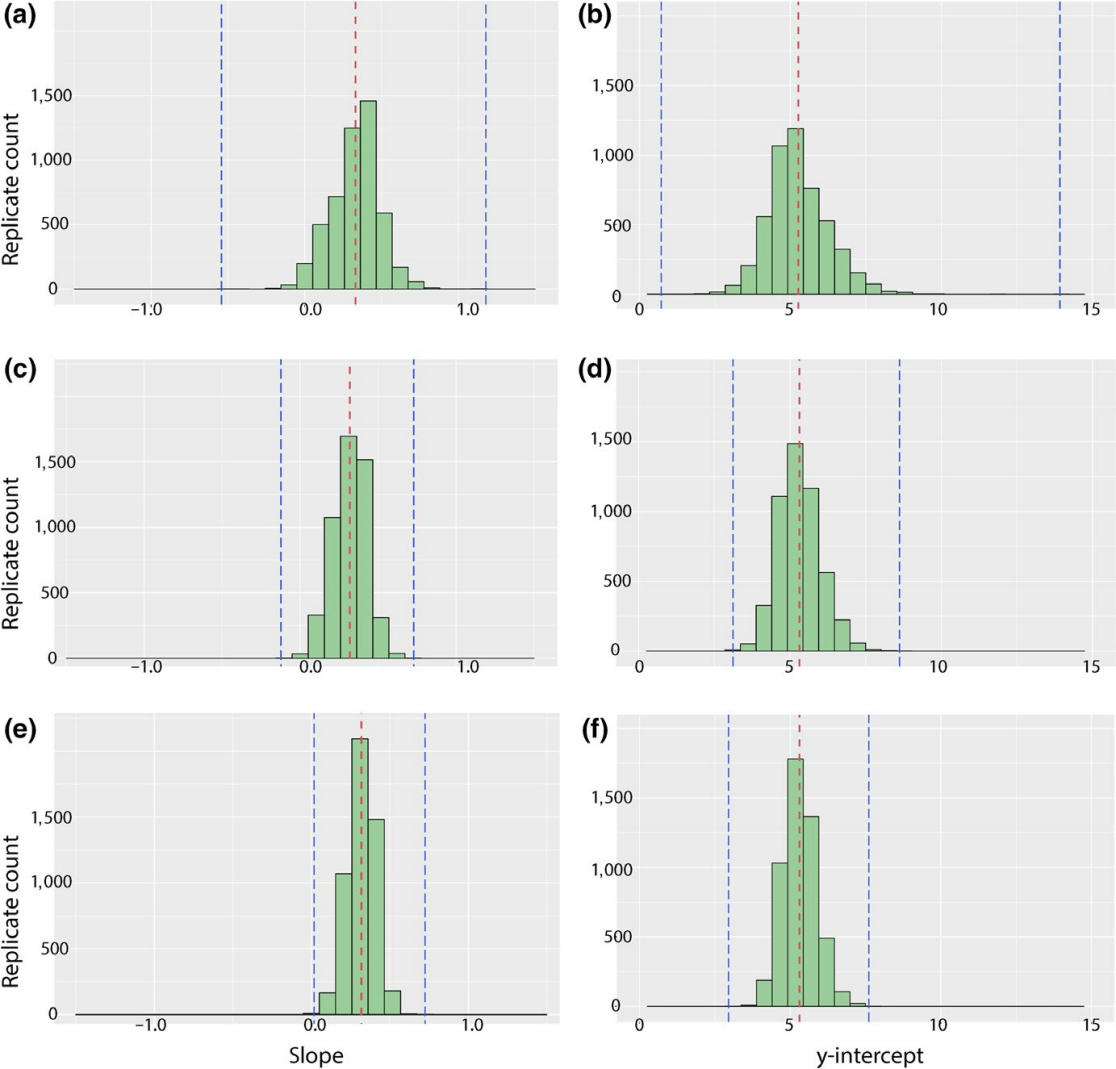
Histograms showing effect of sample size on variability in estimates of biological variables (growth rate and size at birth) as proxied by parameters of the linear model (slope and *y*‐intercept). (a) Distribution of slope values with sampling bin of 10. (b) Distribution of y‐intercept values with sampling bin of 10. (c) Distribution of slope values with sampling bin of 20. (d) Distribution of y‐intercept values with sampling bin of 20. (e) Distribution of slope values with sampling bin of 30. (f) Distribution of *y*‐intercept values with sampling bin of 30. Red lines demarcate the mean; blue lines demarcate the minimum and maximum values

The results of the sensitivity analysis are presented as histograms showing the distribution of values of the 5,000 iterations of each analysis in Figures 4 and 5. As annotated in the figures, the ranges of the maximum and minimum values for the two model parameters of a simple linear model (*y*‐intercept, slope) and that of the coefficient of determination (*R* ^2^) become increasingly narrow and approach a normal distribution with increased sample size. Values for the slope (i.e., growth rate) could be negative, a biologically implausible scenario based on a typical trend of increasing size throughout maturation in tetrapods (but see the paradox frog for an unusual counterpoint in amphibians). These iterations typically recovered a y‐intercept (initial femur size) larger than that of any specimen sampled in this analysis.

## 4 DISCUSSION

### 4.1 Comparisons with other amphibamiforms

#### 4.1.1 Interpretation of double LAGs

Double LAGs are rarely identified in temnospondyls (e.g., McHugh, 2014; Sanchez, Steyer, et al., 2010) but they are not uncommon in lissamphibians (e.g., Castanet & Caetano, 1993; Castanet & Smirina, 1990; Francillon‐Vieillot, Arntzen, & Géraudie, 1990; Smirina, 1994). The interpretation(s) of this pattern in extinct tetrapods draw heavily on work regarding extant tetrapods and frequently propose that this feature represents two cessations in growth during 1 year (aestivation and hibernation) due to unfavorable conditions, such as climatic harshness or fluctuations in prey density. The only other well‐studied example of double LAGs in dissorophoids occurs in the aquatic European branchiosaurid *Apateon.* Sanchez, Steyer, et al. (2010) proposed that climatic fluctuations in the Saar‐Nahe Basin in Germany resulted in the formation of double LAGs in various species and populations of this taxon. This interpretation was based on skeletochronological study of the extant marbled newt (*Triturus marmoratus* Latreille, 1800) across an elevational gradient by Caetano, Castanet, and Francillon (1985) and Castanet and Caetano (1993). The latter authors found a correlation between increased presence of double LAGs in higher elevation populations of *T. marmoratus*, and Sanchez, Steyer, et al. (2010) made a similar correlation for *Apateon* based on inferred paleoaltitude. However, it is important to note that double LAGs should not be considered as a reliable proxy for paleoaltitude. The Richards Spur locality represents a local topographic high (Donovan, 1986), rather than a high‐elevation environment like in the Saar‐Nahe Basin. Furthermore, among extant lissamphibians, double LAGs can occur (albeit more rarely) at relatively low altitudes (e.g., Guarino, Lunardi, Carlomagno, & Mazzotti, 2003; Miaud, Joly, & Castanet, 1993) and double LAGs do not always occur in individuals living in upland or high‐elevation environments (e.g., Eden, Whiteman, Duobinis‐Gray, & Wissinger, 2007; Esteban, Sánchez‐Herráiz, Barbadillo, & Castanet, 2004; Seglie, Roy, & Giacoma, 2010). Lastly, it has been alternatively suggested that the double LAG pattern observed in *Apateon* might actually be related to fluctuations in salinity if the Saar‐Nahe Basin was not situated at a high paleoaltitude but was instead with a close connection to marine environments (e.g., Laurin & Soler‐Gijón, 2010; Schultze, 2009:163, and references therein). It is unlikely that salinity would have drastically affected the growth of the terrestrial *Doleserpeton*, at least postmetamorphosis (if such a transformation occurred), given the ubiquity of the double LAG pattern across the sample and in individuals of probably an “adult” age. However, the potential for other environmental factors to produce essentially indistinguishable double LAG patterns in other taxa emphasizes that the presence of such a pattern cannot be considered to be an alternative proxy for determining whether a population resided at elevation.

In general, it is likely that the full range of variables that may produce double LAGs is related to broader environmental conditions that may occur in many geographic regions and at a range of elevations. Temperature fluctuations, water availability and conditions, and prey density are all potential explanators that can be influenced by climate patterns and that in turn affect tetrapod growth (e.g., Guarino & Erismis, 2008). For example, studies of Couch's spadefoot toad (*Scaphiopus couchii* Baird, 1854) from Arizona revealed that fluctuations between single and double LAGs in single individuals were correlated with fluctuations of monsoonal precipitation (Tinsley & Tocque, 1995), a factor that could have also been influential at low‐latitude paleoenvironments of the early Permian such as Richards Spur (e.g., Woodhead et al., 2010 and references therein). Cessations in growth due to insufficient nutrition may also occur and are usually identified as LAGs with an incomplete circumference, or false LAGs, in extant lissamphibians (e.g., Castanet & Smirina, 1990; Hemelaar, 1985; Sagor, Ouellet, Barten, & Green, 1998), but these were not identified in this sample. The prevalence of double LAGs in our sample is thus interpreted to reflect a recurring and predictable environmental factor that modulated the growth patterns of *Doleserpeton*, particularly because the sample is virtually assured to not represent a single population. Such a factor could include harsh seasonal conditions that, although highly variable within a year (e.g., hot summers and cold winters), were less variable between years, recurring in a similar fashion year‐over‐year. Whether this resulted in a direct physiological effect (e.g., heat stress) or secondary effects (e.g., stressors affecting prey availability) is unknown. The presence of only single LAGs in a few specimens may reflect periods of relative stability on multi‐year scales. Mixed LAG patterns would thus represent fluctuations in environmental conditions. The pattern of double LAGs early in development and single LAGs later in development (seen also in *Apateon*; Sanchez, Steyer, et al., 2010) could specifically represent a diminished need to slow growth once relative maturity was reached, but this pattern is not documented in extant taxa and remains to be explored. A unidirectional transition from climatic instability to climatic stability could be another explanator. The only other terrestrial amphibamiform to be histologically studied is *M. stowi*, the only dissorophoid from either Gondwana or from the Mesozoic. McHugh (2015) proposed that the absence of LAGs in *M. stowi* reflected that the animal grew so fast that it achieved a relatively large body size within the first year. This in turn is suggestive of conditions that would necessitate rapid skeletal maturation, such as pronounced seasonality and climatic harshness that would require rapid, opportunistic growth during the more favorable periods in advance of more adverse conditions. However, additional sampling of terrestrial amphibamiforms will be necessary to better evaluate the disparate histology and inferred life history due to the geographic and temporal occurrence of *M. stowi* from the Permo‐Carboniferous taxa.

Another hypothesis that merits brief discussion is the potential that some of the Richards Spur tetrapods may have resided within part of the karst system, perhaps at the mouth of the cave—this could have served as a local refuge during unfavorable environmental conditions. It could be predicted that such a lifestyle would be captured in the skeletochronology of *D. annectens* if this were true. Unfortunately, the skeletochronology of extant cave‐dwelling lissamphibians has not been extensively explored. Sampling of the subterranean spring salamander (*Gyrinophilus porphyriticus* Green, 1827) by Bruce and Castanet (2006) revealed poorly resolved or entirely absent LAGs, which the authors correlated with reduced seasonality and thus a more constant activity level over the year. Conversely, sampling of surface‐ and cave‐dwelling populations of the Pyrenean newt (*Euproctes asper* Dugès, 1852) by Miaud and Guillaume (2005) showed regular LAG patterns in both populations, which the authors suggested could relate to cessation of growth associated with reduced feeding (of uncertain relationship to reduced prey populations). Although climatic fluctuations may strongly influence prey populations (probably arthropods in the case of *Doleserpeton*), environmental perturbation is not the only limiting factor on prey density. A more comprehensive survey of the skeletochronology and inferred life histories of the Richards Spur tetrapods will be necessary to more thoroughly explore this hypothesis.

#### 4.1.2 Skeletochronology

Our analysis produces a widely variable dataset that cannot be well‐approximated by mathematical growth models, although there is a clear degree of correlation between inferred age and femur size (Table 2; Figure 3). Typically, skeletochronological studies of extant lissamphibians have used either linear or von Bertalanffy (VBGM) models to attempt to fit their data to a predictable growth curve. In this study, it was not possible to fit a VBGM to the data, perhaps because of the relative scaling between the range of sizes among femora and the range of inferred ages in the analysis. The VBGM predicts a rapid growth rate in early ontogeny that plateaus in later stages, and thus, incomplete sampling of the growth trajectory may produce a dataset that cannot be properly modeled using nonlinear models. It is worth noting that the VBGM was originally developed for teleost fish (von Bertalanffy, 1938) and may thus be an inherently limited or poorly applicable model for most tetrapods, either extant or extinct. The spread of our data and its relatively poor fit to various models could be regarded as an artifact of relatively low sample size when compared to many studies on extant lissamphibian development, which may sample over a hundred individuals. Conversely, it may also be attributed to natural variation and plasticity in *D. annectens* that is only detected through our large sample size and sampling of comparably sized (but evidently variably aged) individuals. Developmental plasticity is a widespread ecological attribute in extant lissamphibians (e.g., Alcobendas & Castanet, 2000; Augert & Joly, 1993; Denoël & Joly, 2000; Eden et al., 2007) that permits them to adapt to local environments and that in turn produces variation in growth curve reconstructions. This hypothesis can be extended to *D. annectens* based on the evidence from the double LAG pattern that seasonal conditions were pronounced and disparate. It must also be considered that a pattern of microevolution may be confused for a pattern of plasticity when analyzing time‐averaged populations (see commentary by Schoch, 2014:519–520). It is also important to note that even in the absence of a directional microevolution (e.g., increased body size), time‐averaging is still likely to produce variation that exceeds that of any single population because it samples multiple populations. The sample analyzed here undoubtedly represents a time‐averaged assemblage due to the nature of the karst deposits based on the absolute age constraints (289–286 Ma) recovered by previous analyses of speleothems at Richards Spur (MacDougall et al., 2017; Woodhead et al., 2010). These constraints are fairly narrow for an extinct taxon, which suggests that a hypothesis of plasticity has considerable merit, but three million years is still a considerable amount of time in absolute terms. Additional work on other amphibamiforms and temnospondyls with a high degree of stratigraphic and temporal control will be needed to further evaluate our hypothesis.

#### 4.1.3 Effects of sample size on growth curve reconstruction

The subsampling experiments that we performed here indicate that growth curve reconstruction is extremely susceptible to low sample size. Some permutations of the smallest subsampling (*n* = 10), a common sample size for extinct tetrapods (see Steyer et al., 2004, for a temnospondyl example), could be fit with a simple linear model that produced a correlation coefficient of nearly 1.0 (Figure 4). This would suggest a very strong correlation between inferred age and skeletal size (of the femur). However, in our analysis, this result was rare compared to the opposite extreme, a fit of 0.000, or no correlation. Similar extremes could be noted for the slope and the y‐intercept values (representing growth rate and femur length at birth, respectively; Figure 5). In subsamples of *n* = 10, slopes could be negative, zero (horizontal line), or approaching infinity (nearly vertical line), all of which are implausible for tetrapods. There is only one well‐known example of an extant lissamphibian which decreases in size throughout ontogeny—the aptly named paradox frog (*Pseudis paradoxa* Linnaeus, 1758; Emerson, 1988). This diminution occurs during metamorphosis when much of the skeleton remains unossified and would thus not be reflected in the long bone histology, and the disparity between larval and adult sizes is probably related to an unusually large tadpole stage that evolved in response to environmental harshness (Emerson, 1988). In a similar vein, a few replicates with a subsample of *n* = 10 produced y‐intercept values that greatly exceeded the size of all specimens sampled in this analysis.

Sample sizes are typically low for any extinct tetrapod because of taphonomic biases, incompleteness of specimens, and restricted access to materials for destructive sampling. As a result, these datasets are less robust than those that can be produced for extant tetrapods, especially lissamphibians, for which several hundred individuals may be sampled by using relatively noninvasive methods such as toe clippings. The few studies that have previously produced a growth curve for temnospondyls (e.g., Konietzko‐Meier & Sander, 2013; Steyer et al., 2004) have sampled relatively few (<12) specimens but also produced a well‐fit curve (*R* ^2^ > .90) using linear models (either simple linear or second‐order polynomial). Although this may be interpreted as reflective of low intraspecific variation throughout ontogeny, such results may also be interpreted as an artifact of low sampling size or incomplete sampling of the entire ontogenetic trajectory (i.e., only a stage that is well‐approximated by linear models) based on our findings. A strong fit is not equivalent to biological accuracy. This is especially salient if it has not been tested whether multiple specimens of the same size are of the same predicted maturity. Typically, paleohistological studies of growth have tried to sample as broad of a size range as possible, but this often results in large gaps in size between specimens and an absence of testing of multiple specimens of a comparable size, which is arguably the more direct way to assess whether size and age are tightly correlated.

These findings are not meant to suggest that we interpret well‐fit linear models like those of Steyer et al. (2004) and Konietzko‐Meier and Sander (2013) to represent an extremely unlikely outlier or to be biologically implausible. Linear growth may occur during part of a growth trajectory and may thus reflect only incomplete sampling of the ontogenetic range. Furthermore, the results of our study on *Doleserpeton* (a miniaturized, terrestrial early Permian amphibamid) are not directly comparable to those on *Metoposaurus* von Meyer, 1842 and *Dutuitosaurus* Dutuit, 1976 (large, paedomorphic, obligately aquatic Late Triassic metoposaurids). More work is necessary to determine whether there is a disparate rate of variation in ontogeny between ecologies and body sizes and across temnospondyl clades (e.g., Schoch, 2014; Steyer, 2000; Witzmann, Scholz, & Ruta, 2009). Neither is this a criticism of previous workers. The inherent limitations of paleohistological work confound a sample size comparable to that of this study for the vast majority of extinct tetrapods. Our own sample, although much larger than typically achievable, is inherently a microcosm in the same vein as other studies in examining the paleobiology of taxa that may have survived for millions of years through a sample that is several orders of magnitude smaller.

However, our unusually high sample does provide a cautionary tale showing that limited sample size and limited sampling of specimens of the same size (to test whether they are the same age) can produce misleading or biologically implausible results. Regardless of whether a sample size for a paleohistological study is considered to be standard for the field, this does not negate the fact that interpretations derived from low sample size are inherently tenuous. Obtaining a strongly fit curve (or line) to a dataset compiled from a low sample size should be treated with some skepticism because other large datasets examining ontogeny in extinct tetrapods (e.g., Griffin & Nesbitt, 2016; Sander & Klein, 2005) often recover patterns that are indicative of marked plasticity and for which growth curves cannot be well‐modeled. Linear models in particular are not typically regarded as accurate or precise models for growth in tetrapods because growth in the form of size changes is virtually assured to plateau at later life stages (although linear models may approximate particular stages of growth). Assessing the effects of methodology, such as sample size, sampling bins, and bin frequency is an important step in evaluating previous growth curve reconstructions. Modern lissamphibian studies have long shown a poor correlation between age, either inferred or known, and body size in some taxa (e.g., Halliday & Verrell, 1988; but see Laurin & Germain, 2011), regardless of whether factors such as number of sampled populations, uniform time of sampling, and biological sex can be controlled for. Better correlations are produced between body mass and body size, but the former is not available to paleontologists and is difficult to produce without relying heavily on either age (poor correlation) or body size (usually unknown and the dependent variable in this relationship). In most instances of paleontological studies, it cannot be demonstrably proven that all of the individuals in a given locality were part of the same interbreeding biological population, rather than a time‐averaged assemblage. However, the fissure fill nature of Richards Spur possibly exacerbates the time‐averaging relative to other localities, and this should be considered when comparing our findings to those of other studies.

#### 4.1.4 The role of apomorphy‐based identification in histology

Apomorphy‐based identification (e.g., Bell, Gauthier, & Bever, 2010; Nesbitt & Stocker, 2008) is a taxonomic practice that relies on unique derived features (apomorphies) to justify taxonomic identifications and specimen referrals. It is considered to be more rigorous than resemblance‐based identification but can also be limited by outdated taxonomy. Apomorphy‐based identification is rarely applied for paleohistological work, which is the product of a number of factors related to histological methods. First, the majority of all paleohistological work to date focuses on isolated postcranial elements, which are more readily accessible but also less likely to be properly referable. Second, a historic precedent on cranial characters and features for identification and diagnoses of new taxa limits the ability to link isolated postcrania with the more diagnostic crania without articulated specimens. Thus, taxonomic identification of many specimens selected for histological sampling is identified by a combination of resemblance‐based identification and circumstantial evidence, such as stratigraphic occurrence and faunal community assemblage. We are not suggesting that this is a poor practice—in many instances, it is both robust and the only viable practice—but that does not eliminate the limitations associated with data obtained through a sample selected in this way, and such shortcomings should be explicitly stated for transparency.

With respect to this study, *D. annectens* is only one of three amphibamiforms and nine dissorophoids at the Richards Spur locality. Because of the size disparity between adult amphibamiforms and adult olsoniforms, small yet relatively well‐ossified elements can be excluded from consideration as markedly immature olsoniforms. This assumption is further validated upon examination of the data, as there are no sampled specimens of a markedly young age. However, the other two amphibamiforms, *P. mayi* (Fröbisch & Reisz, 2008) and *T. dolesensis* (Anderson & Bolt, 2013), are represented almost exclusively by cranial material. More broadly, little is known about terrestrial Permian amphibamiform postcranial anatomy, as many taxa are represented only by cranial material, and what is known indicates little more than slight variation in proportions between species (e.g., Clack & Milner, 2010; Daly, 1994). Konietzko‐Meier et al. (2016) have noted that morphologically similar long bones of other early Permian tetrapods (sampled from the Briar Creek locality) can exhibit several distinct histotypes, and a conserved morphology in many postcranial elements may characterize each of the terrestrial dissorophoid clades owing to their shared lifestyle (e.g., Gee & Reisz, 2018). Based on the available data, *D. annectens* was markedly smaller than *P. mayi* and slightly smaller than *T. dolesensis* and was far more abundant than both at Richards Spur. The longstanding interpretation of *D. annectens* being exceptionally abundant at Richards Spur (e.g., Bolt, 1969) precedes the more recent discoveries of the other, rare amphibamiform taxa. Without postcrania of *P. mayi* and *T. dolesensis,* isolated amphibamiform postcrania cannot be confidently excluded from belonging to one of these taxa and not to *D. annectens*. Thus, while it is accurate to state that amphibamiform material is very abundant at the site, relative abundances of the various amphibamiforms are more difficult to elucidate. Although cranial material is not the best proxy for assessing relative abundance, it is the only semi‐reliable one given the historic precedent on distinguishing taxa by cranial features. Cranial material confidently referable to *D. annectens* is more abundant than that of either *P. mayi* (represented by three skulls from the locality) or *T. dolesensis* (represented by the holotype, a skull; Anderson & Bolt, 2013; Fröbisch & Reisz, 2008). We would thus predict that even if there is inadvertent capture of non‐*Doleserpeton* amphibamiforms in our sample, the proportion of “foreign” taxa would be relatively minimal and thus unimportant from a statistical perspective. It is likely that such capture would increase the observed variation under an assumption that the three amphibamiforms did not follow an identifical growth trajectory or achieve the same maximum body size. However, without a clearer understanding of various attributes of the ontogeny of *P. mayi* and *T. dolesensis* (e.g., maximum body size), it is not clear exactly how capture of these taxa in our sample could influence the interpretations of the data.

The challenge of working with isolated materials is not limited to this study, but we reiterate that it is important to be explicit about this shortcoming, nonetheless. Paleohistological sampling is fundamentally opportunistic. Limitations on accessibility to specimens for histological sampling of extinct tetrapods typically result in the near‐exclusive use of isolated elements that are identified by circumstantial evidence (e.g., stratigraphic occurrence in a monotaxic bone bed, relative abundance) or resemblance‐based identification. Outside of mass‐death assemblages that probably represent a single catastrophic event (e.g., the metoposaurid *Dutuitosaurus ouazzoui* Dutuit, 1976; Steyer et al., 2004), the sampled population usually cannot be determined to represent a true, single, original population. The potential problem of inadvertently sampling other taxa is not exclusive to this study (e.g., Konietzko‐Meier & Klein, 2013). In this study, we followed previous practices and identified femora that clearly matched those identified to *D. annectens* in past studies (e.g., Sigurdsen & Bolt, 2010) and have observed larger dissorophoid femora from the locality that are too large to belong to *D. annectens* but that are too small and well‐ossified to belong to the co‐occurring olsoniforms. Histological variation that would clearly indicate divergent growth patterns attributable to taxonomy is not present in our sample beyond some variability in LAG patterns. This is not itself evidence for explicit variability associated with taxonomy based on our previous discussion regarding the formation of double LAG patterns.

## 5 CONCLUSION

Here, we have presented a skeletochronological analysis of the presumed stem lissamphibian *D. annectens*, represented by the largest sample size for a histological study of a Paleozoic tetrapod to date. Our analysis reveals a high degree of variability within the sample, likely reflecting developmental plasticity, a common ecological strategy among extant lissamphibians to cope with unstable and unpredictable environmental conditions. These findings thus suggest the retention of a deep temporal origin of a life‐history strategy common to many metazoan clades that characterizes both terrestrial amphibamiforms and extant lissamphibians and that may have contributed to the persistence of this particular clade of temnospondyls. The presence of double LAGs in most of our specimens further supports this hypothesis, indicating that these animals were often slowing their growth twice a year and thus experiencing two distinctly seasonal types of climatic harshness. Poor correlation between inferred age (proxied by LAG count) and body size (proxied by femur length) is suggestive of developmental plasticity that would be adaptive for the climate conditions at Richards Spur, although microevolution, time‐averaging, and inadvertent capture of other amphibamiforms cannot be fully excluded.

Low sample size inherently undermines the reliability of statistical inference (e.g., Button et al., 2013). The results of our sensitivity analysis have implications specifically for similar skeletochronological studies of extinct tetrapods because they indicate that low sample size may mask variation and produce artificial results that can be highly improbable (e.g., negative growth rates) and highly compelling (well‐fit curves). Low sample size is a reality of working with the fossil record, and our results should not be considered as a criticism of previous studies that utilized lower sample sizes. However, our analysis underscores the point that caution should be exercised in making interpretations from growth curves reconstructed from low sample sizes, even if a well‐fit curve is recovered from the available data. This may differ between clades with so‐called determinate (e.g., birds, mammals) and indeterminate (e.g., lissamphibians) growth, as well as disparate life strategies that affect ontogeny and survivorship within a clade. Simply because a sample size is “normal” or relatively high for a paleontological study does not negate the fact that when the sample size is small in absolute or statistical terms, it may be lacking in controls (e.g., stratigraphic), and drawing inferences from such a sample cannot be considered to be robust. This should not preclude the forming of evidence‐based interpretations and informed speculation but rather emphasizes the need for limitations to be made explicit and for workers to avoid making excessive extrapolations in their conclusions from the available limited data.

## CONFLICT OF INTEREST

None declared.

## AUTHOR CONTRIBUTIONS

BMG and RRR conceived and designed the study. RRR contributed materials. BMG performed the data analyses. BMG and YH interpreted the data and drafted the manuscript. All authors contributed to editing the manuscript and approved it for submission.

## Figures and Tables

**Figure A1:**
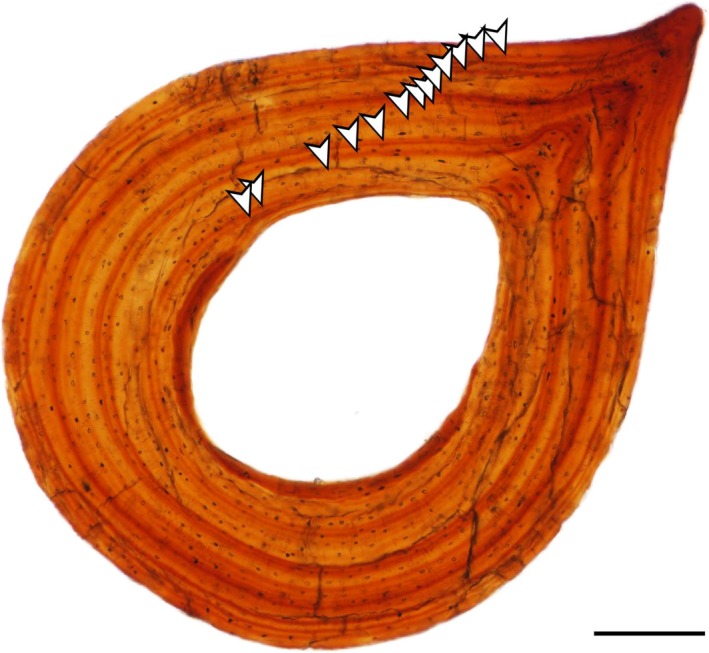
Microphotograph of ROMVP 80676. Scale bar = 0.25 mm

**Figure A2:**
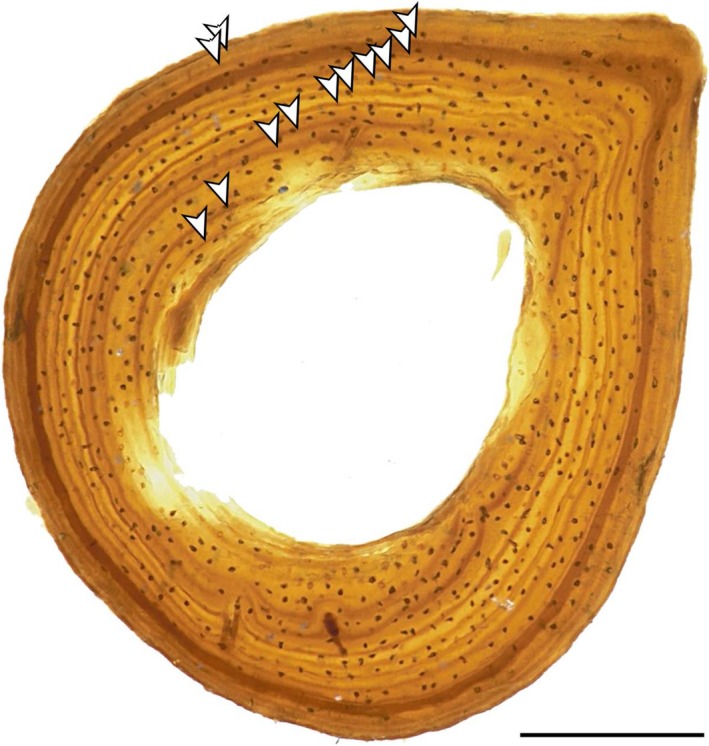
Microphotograph of ROMVP 79319. Scale bar = 0.25 mm

**Figure A3:**
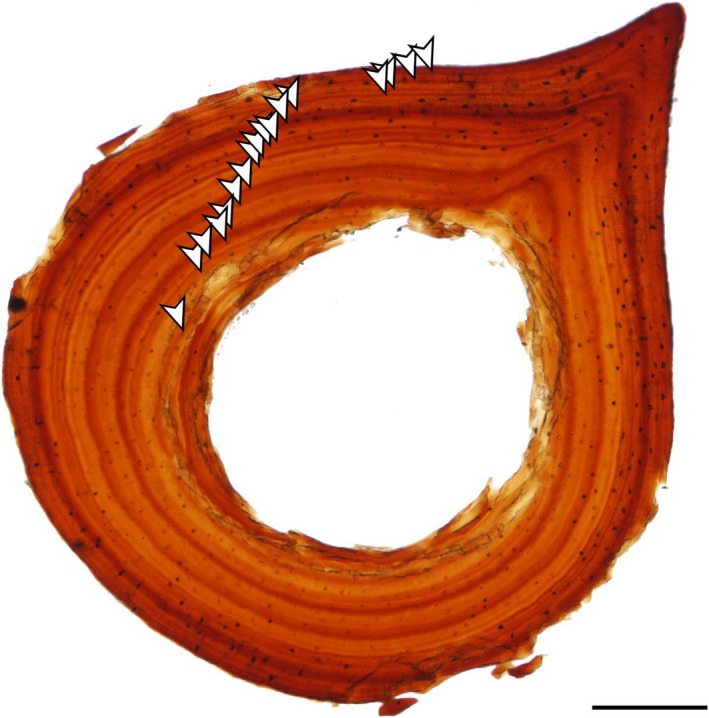
Microphotograph of ROMVP 79337. Scale bar = 0.25 mm

**Figure A4:**
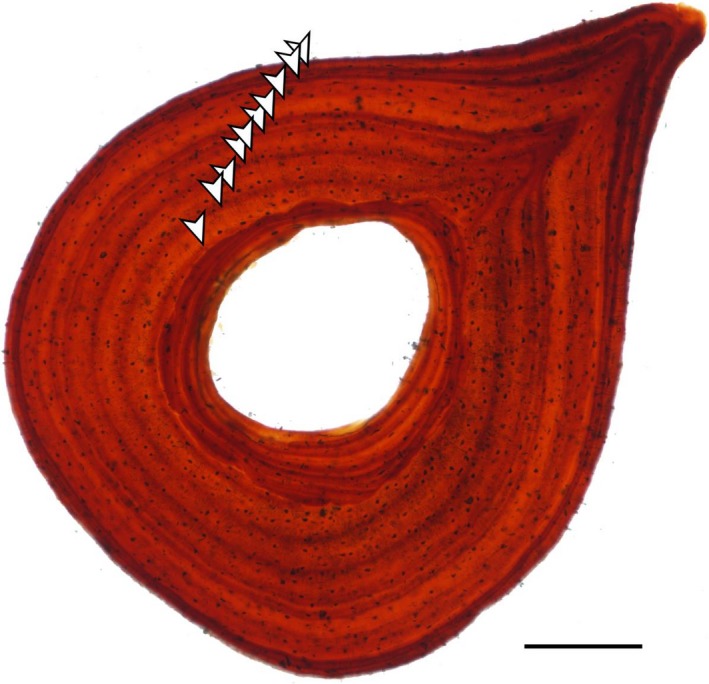
Microphotograph of ROMVP 80680. Scale bar = 0.25 mm

**Figure A5:**
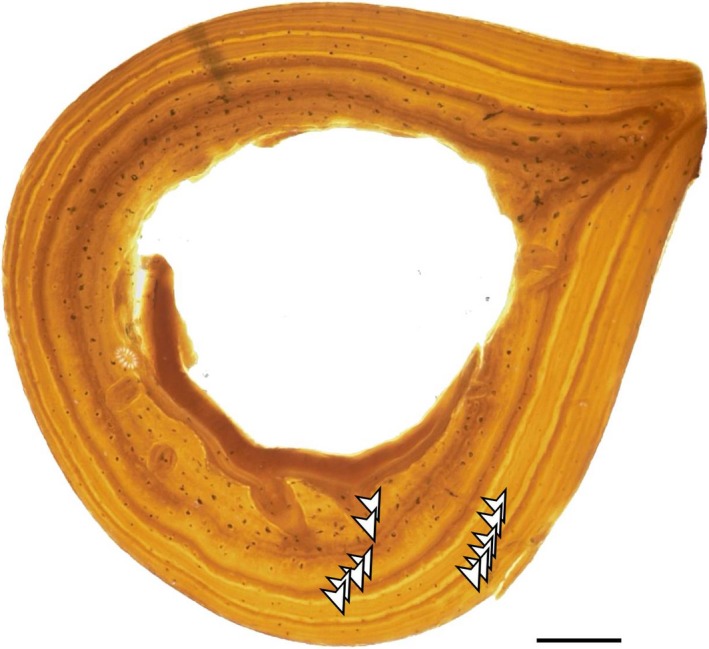
Microphotograph of ROMVP 79330. Scale bar = 0.10 mm

**Figure A6:**
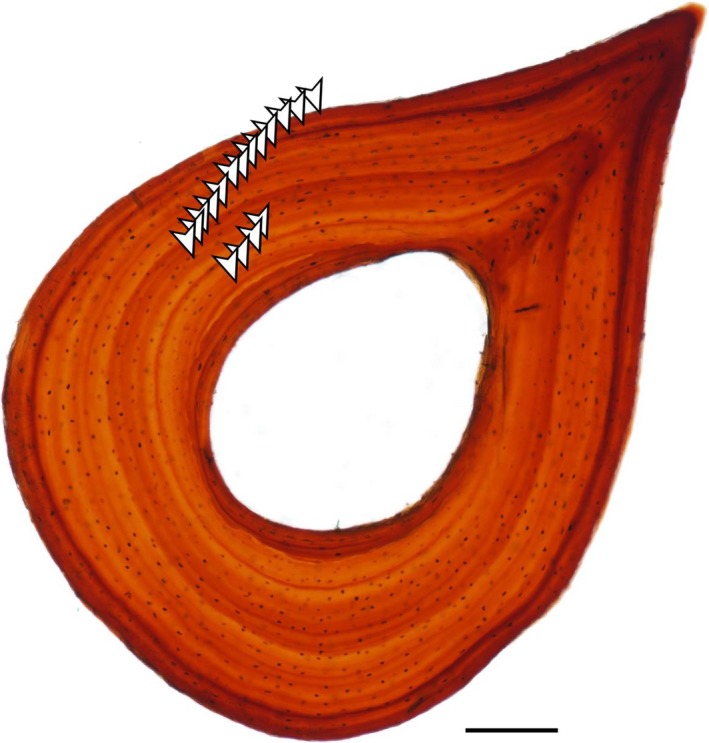
Microphotograph of ROMVP 80667. Scale bar = 0.10 mm

**Figure A7:**
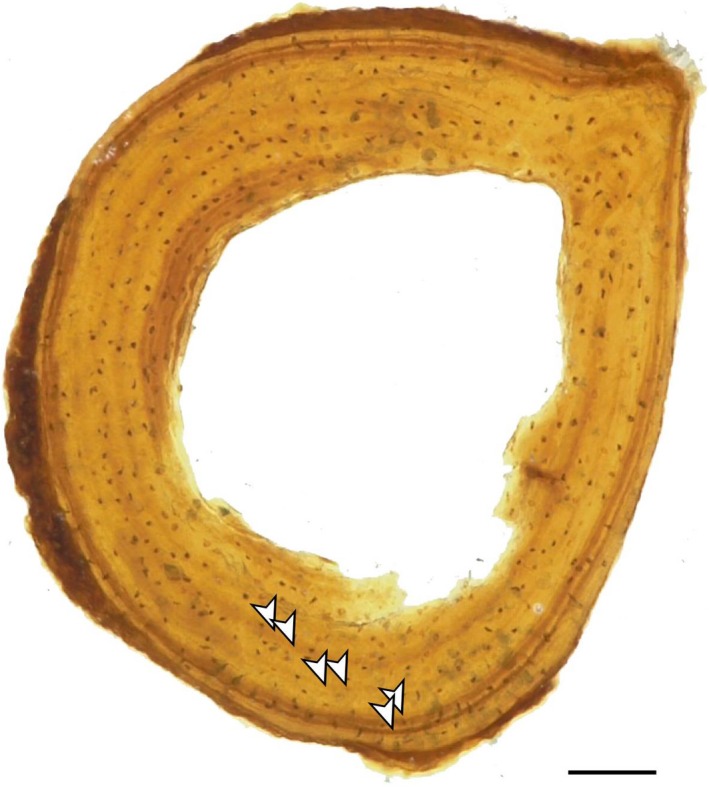
Microphotograph of ROMVP 79331. Scale bar = 0.10 mm.

## Data Availability

All data included in this study are included in the main document. Thin sections are available to qualified researchers at the Royal Ontario Museum (ROMVP), Toronto, ON, Canada.
