# Supplementary figures and images for: Histological skeletochronology indicates developmental plasticity in the early Permian stem lissamphibian Doleserpeton annectens
Source: Ecol Evol. 2020 Feb 6;10(4):2153–69. doi: 10.1002/ece3.6054 (PMC7042763; doi:10.1002/ece3.6054)

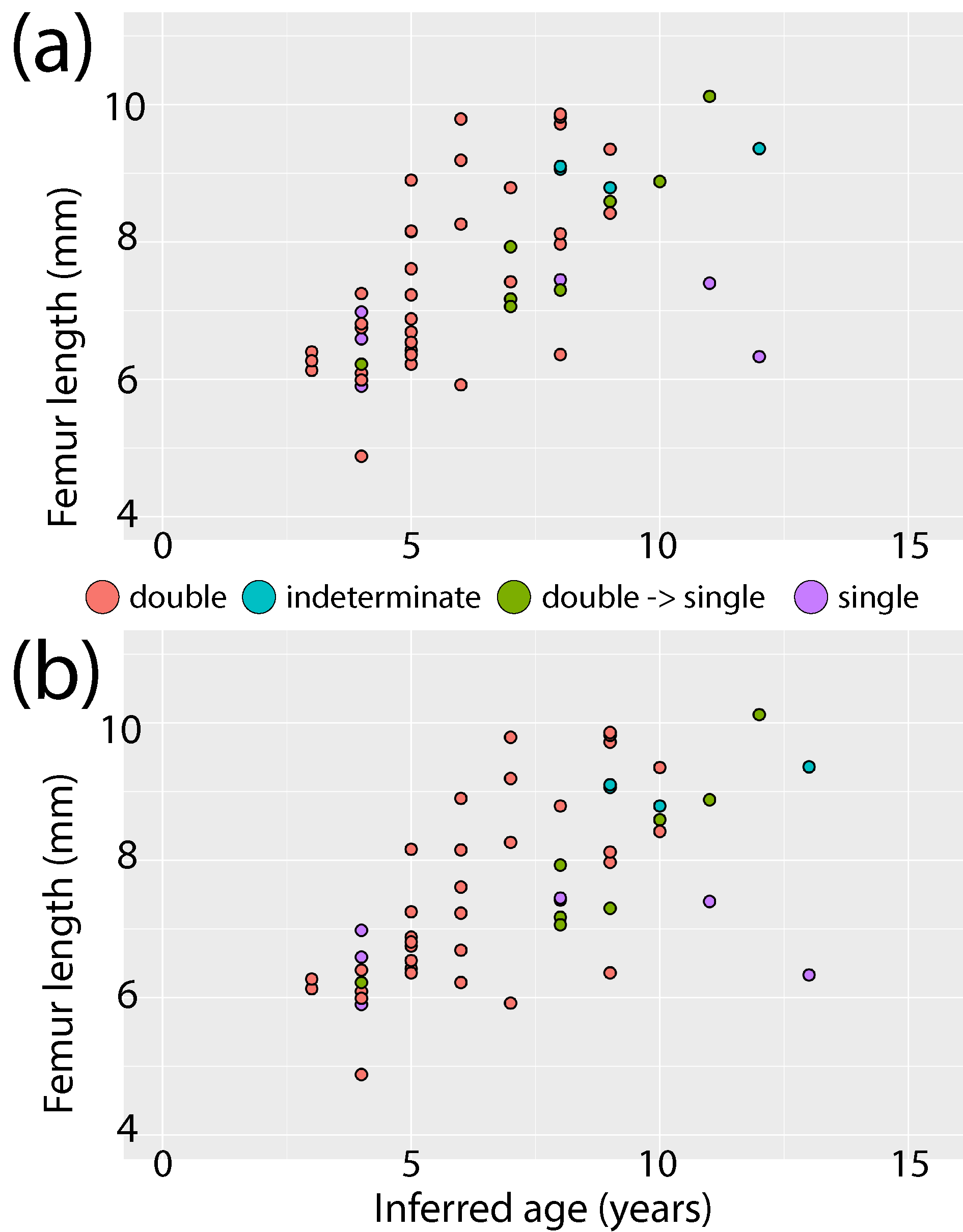

Supplement: Supplementary file 1 [file ECE3-10-2153-s001.tif]
