# Supplementary material for: Histological skeletochronology indicates developmental plasticity in the early Permian stem lissamphibian Doleserpeton annectens
Source: Ecol Evol. 2020 Feb 6;10(4):2153–69. doi: 10.1002/ece3.6054 (PMC7042763; doi:10.1002/ece3.6054)
Supplement: Supplementary file 2 [file ECE3-10-2153-s002.docx]

**Figure S1. Data plots mapped by the type of observed LAG pattern. (a)** Data plot of age inferred from observational counts (raw data) versus femur length. **(b)** The same inferred from retrocalculated counts (adjusted data).
